# Decomposing drivers of air pollutant emissions in China: A hybrid LMDI and Geographically Weighted Regression approach

**DOI:** 10.1371/journal.pone.0333898

**Published:** 2025-10-21

**Authors:** Bo Zhang, Yijing Liang

**Affiliations:** 1 Environmental Technology Division, Universiti Sains Malaysia, Penang, Malaysia; 2 National Key Laboratory of Lubricating Materials, Lanzhou Institute of Chemical Physics Chinese Academy of Sciences, Lanzhou, China; Agricultural Sciences and Natural Resources University of Khuzestan, IRAN, ISLAMIC REPUBLIC OF

## Abstract

Air pollution control is an urgent problem in the field of environment, and it is crucial to accurately identify emission driving factors and collaborative emission reduction paths. In order to construct and analyze the driving mechanism of atmospheric pollutant emissions and explore the potential for regional collaborative emission reduction, an innovative three-stage progressive analysis framework was developed by combining Logarithmic Mean Divisia Index (LMDI) decomposition and Geographically Weighted Regression (GWR), which includes factor decomposition, spatial modeling, and collaborative optimization. Through empirical analysis, it was found that the energy intensity effect in Tangshan city reduces emissions by an average of −14.834 million tons per year, becoming the core driving force. The synergistic emission reduction ratio of SO2-PM2.5 in the Beijing Tianjin Hebei region reached 1: 0.38, with an average annual emission reduction of 297000 tons and a regional synergy index of 0.85 (*p* < 0.01), significantly better than other pollutant combinations. The adjusted R2 of the GWR model reached 0.86, the residual Moran’s I index was 0.07, and the proportion of significant variables reached 75%, which is 15.28% higher than other models. In addition, the Akaike information criterion corrected by the GWR model was reduced by an average of 12.78% compared to other models. The results indicated that the synergistic effect of multi factor decomposition and spatial heterogeneity analysis could significantly enhance the regional adaptability of emission reduction strategies, providing scientific support for cross regional collaborative governance.

## 1. Introduction

With the acceleration of global industrialization, air pollution and climate change have become dual challenges threatening human sustainable development. The combustion of fossil fuels in fields such as electricity, industry, and transportation emits large amounts of atmospheric pollutants such as CO2, NOx, and particulate matter, leading to increasingly prominent regional composite pollution problems [1,2]. Research has shown that atmospheric pollutants and greenhouse gas emissions have the same source, and their synergistic reduction has the potential to achieve the “dual carbon” goals and improve air quality [3]. However, traditional emission reduction (ER) strategies often focus on a single pollutant or industry, lacking systematic research on collaborative ER pathways for multiple pollutants, resulting in high policy implementation costs and low efficiency [4]. The existing research has made significant progress in methodology, and the Logarithmic Mean Divisia Index (LMDI) decomposition has been widely used in the analysis of carbon emission (CE) drivers due to its advantages of no residuals and reversible decomposition. However, most studies focus on a single dimension of energy structure or economic activity, and there is little exploration of the cross sectoral impact mechanism of pollutant collaborative ER [5]. The Geographically Weighted Regression (GWR) model, by introducing spatial heterogeneity analysis, can reveal the spatial distribution characteristics of pollutant emissions and their correlation with factors such as economic development and population size. However, existing research mostly focuses on the field of CEs, and there is insufficient analysis of the geographic spatial characteristics of collaborative ER of atmospheric pollutants [6]. In response to the above issues, a three-stage analysis framework integrating LMDI decomposition and GWR is proposed. Based on panel data of prefecture level cities, the framework progresses layer by layer from factor decomposition, spatial modeling to path optimization. The study selected SO₂, NO_x_ and PM_2.5_ as the main analysis objects, based on the following considerations: Firstly, in the research on air pollution control in China, SO_2_, NO_x_, and PM_2.5_ are the three main pollutants with significant health and environmental impacts. SO_2_ and NO_x_ are the main precursors of acid rain and photochemical smog, posing a direct threat to respiratory and cardiovascular health; PM_2.5_, as a core indicator of particulate matter pollution, is closely related to various chronic diseases and mortality risks. Secondly, SO₂, NO_x_ and PM_2.5_ are often co emitted with CO_2_ during industrial combustion and transportation emissions. Therefore, measures to reduce their emissions can achieve coordinated control of carbon emissions to a certain extent. Once again, the current national pollution prevention and control policies and atmospheric governance goals have clear requirements for the reduction of these three types of pollutants, such as the “Action Plan for Air Pollution Prevention and Control” and local emission standards, which have strong policy operability; Finally, from the perspective of industry and energy structure, industrial production and energy consumption patterns make outstanding contributions to SO₂, NO_x_ and PM_2.5_ emissions. Research on these pollutants can help reveal the correlation between economic activities and air quality improvement, and provide auxiliary references for achieving the “dual carbon” goals [7–9]. The research aims to systematically analyze the temporal driving mechanism and spatial heterogeneity of atmospheric pollutant emissions, identify key paths for regional collaborative ER, and optimize strategy combinations. The innovation of the research lies in constructing a spatiotemporal coupled driving factor analysis system, embedding six LMDI decomposition factors such as emission intensity and energy intensity into the GWR spatial weight matrix, providing theoretical support for formulating scientific and efficient regional air pollution prevention and control policies.

## 2. Related works

In recent years, research on collaborative ER of atmospheric pollutants has emerged, and numerous scholars have proposed various innovative methods from different perspectives to address this complex environmental challenge. J. Cheng et al. proposed a method for jointly deploying air pollution and climate policies to improve air quality and address climate change. By combining emission, atmospheric, and health models, it was proven that measures such as upgrading industrial facilities, stricter vehicle emission standards, and phasing out fossil fuels can achieve ER targets [10]. Y. Li et al. proposed an extended Kaya characteristic and grey relational model to analyze the synergistic effect of air pollutants and CO_2_ reduction in Beijing. By quantifying the collaborative ER effect, it provided reference for Beijing to play a leading role in carbon reduction and pollution reduction [11]. S. L. Nordahl et al. investigated the effects of waste characteristics, pretreatment processes, and composting conditions on atmospheric pollutant emissions in order to clarify the greenhouse gas and air pollutant emissions during the composting process. By analyzing the impact of factors such as waste characteristics on emissions, composting management and ER measures were optimized [12]. L. J. Cushing et al. proposed a method to analyze the correlation between community risk levels and power plant site selection in order to explore the relationship between racism and air pollution source distribution in the United States. By analyzing relevant air pollutant data, the conclusion that racism in the housing market leads to unequal emission burdens from power plants today was revealed [13]. K. Gu et al. proposed an adaptive multi-scale transform domain method that integrates local and global information for monitoring air pollution, providing a new approach for refined pollution management [14]. Y. Zhang et al. studied the interaction between extreme climate events and temperature and wind speed, providing a reference for understanding the impact of meteorological conditions on pollutant diffusion [15]. R. Sun et al. aimed to reveal the impact of extreme daytime temperature range changes on environmental pollution and ecological vulnerability in karst regions worldwide. By characterizing the coupling relationship between extreme daytime temperature range and wind speed, they assessed regional climate risks and pollution diffusion potential, providing a basis for developing differentiated environmental governance and adaptation strategies [16]. In order to enhance the ability to predict air pollution, C. Wu et al. evaluated the current status of artificial neural networks, recurrent neural networks, and hybrid neural network models in predicting air quality, and systematically summarized the improvement directions and future research paths of air quality prediction methods, providing reference for environmental pollution control [17]. Meanwhile, C. Wu et al. systematically reviewed the research progress on PM2.5 prediction from 2014 to 2024, providing comprehensive references for improving the accuracy of air pollution prediction, supporting environmental governance, and public health decision-making [18].

LMDI decomposition, as an effective quantitative analysis tool, is widely used in the study of collaborative ER of atmospheric pollutants and CEs to analyze emission driving factors and ER potential. E. Koilakou et al. proposed a method that combines decoupling and LMDI decomposition analysis to investigate the driving factors of energy and carbon intensity in the United States and Germany. By identifying and arranging factors that define carbon and energy intensity, it was confirmed that energy intensity played a dominant role in CO_2_ emissions [19]. H. Cui et al. proposed a hybrid prediction model that combines LMDI decomposition model and genetic algorithm to promote low-CEs reduction in the construction industry. By analyzing driving factors and optimizing neural networks, accurate prediction of peak and timing of building CEs could be achieved [20]. A. B. Hammamia et al. proposed an analysis method based on logarithmic division decomposition and LMDI decomposition to develop effective CO2 reduction strategies. By analyzing and predicting the main potential factors, a deep understanding of the long-term relationship between CO_2_ emissions and influencing factors was achieved [21].

GWR, as a powerful spatial analysis tool, demonstrates unique advantages in analyzing the complex spatial relationships of regional environmental problems. M. W. Naikoo et al. proposed an analysis method that combines Ordinary Least Squares (OLS) and GWR to analyze and quantify land use changes and their driving factors in suburban areas of the Delhi National Capital Region. By conducting natural land use cover classification and regression analysis, the main driving forces of built-up area expansion and their spatial heterogeneity goals were revealed [22]. A. Comber et al. analyzed standard GWR, mixed GWR, and multi-scale GWR in order to better apply GWR in spatial analysis of social and environmental data. By considering secondary issues such as collinearity, outliers, and error terms, the accuracy and effectiveness of spatial data analysis were promoted [23]. V. Isazade et al. proposed a comprehensive analysis method that combines GWR, OLS, and spatial auto-correlation to analyze the COVID-19 pandemic situation in Iran. ArcGIS was used to analyze confirmed cases and death data, revealing the spatial distribution characteristics and clustering patterns of the epidemic [24].

Based on the above, various research methods have achieved phased results in the field of collaborative ER of atmospheric pollutants. Traditional models and emerging technologies continue to optimize and upgrade in analyzing emission drivers, quantifying synergistic ER effects, and revealing spatial heterogeneity. However, existing methods still have limitations, such as deficiencies in complex interactions of multiple pollutants, cross regional policy coordination, and long-term dynamic prediction. Therefore, the research will focus on building a more systematic and accurate collaborative ER analysis framework, integrating the advantages of LMDI decomposition and GWR models, deeply exploring ER potential, and providing more forward-looking and operable decision support for air pollution control.

## 3. Methods and materials

### 3.1. Construction of a collaborative ER analysis system for air pollutants combining LMDI decomposition and GWR

**Fig 1:**
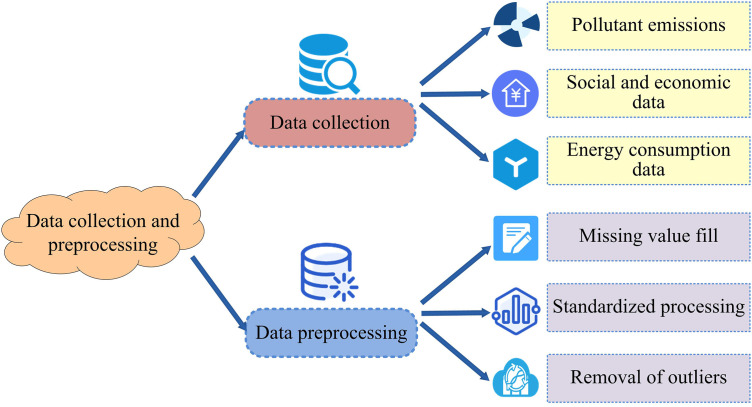
Data collection and preprocessing process.

To systematically analyze the driving mechanism of atmospheric pollutant emissions and identify regional collaborative ER paths, an analysis framework for collaborative ER of atmospheric pollutants combining LMDI decomposition and geographically weighted regression was studied and constructed. This framework is a three-stage progressive analysis framework, mainly divided into factor decomposition stage, spatial modeling stage, and collaborative optimization stage. The factor decomposition analysis process mainly uses the LMDI decomposition method to quantitatively decompose pollutant emissions from multiple factors, in order to clarify the contribution of each factor to emission changes [25]. The spatial regression modeling part uses the GWR model to analyze the spatial heterogeneity of pollutant emission factors between different regions and identify the impact of regional characteristics on ER pathways. The collaborative optimization path is based on factor decomposition and spatial regression results, combined with the interaction between multiple pollutants, to construct a multi-pollutant collaborative ER path selection scheme [26,27]. To ensure the scientificity and operability of the entire analysis process, the study clarified the types and research scope of target pollutants in the early stage of model construction, and carried out data collection and preprocessing work. As shown in Fig 1.

As shown in Fig 1, the study focuses on three typical atmospheric pollutants, SO_2_, NOx, and particulate matter, as the main analysis objects, with the core objective of analyzing the driving mechanism of atmospheric pollutant emissions and focusing on the emission evolution law of atmospheric pollution. The data preprocessing process mainly includes three operations: spatial interpolation, standardization, and missing value completion. In spatial interpolation, the inverse distance weighting method is used to obtain the spatially continuous values of city level units. In terms of indicator standardization, the Gross Domestic Product (GDP) and other inconsistent variables are used as objects, and the minimum and maximum normalization methods are used to improve comparability. For missing data, adjacent annual mean values in the same region are used for interpolation to ensure data continuity and model input integrity [28,29].

### 3.2. Establishment of a model for influencing factors of atmospheric pollutant emissions based on LMDI decomposition

**Fig 2:**
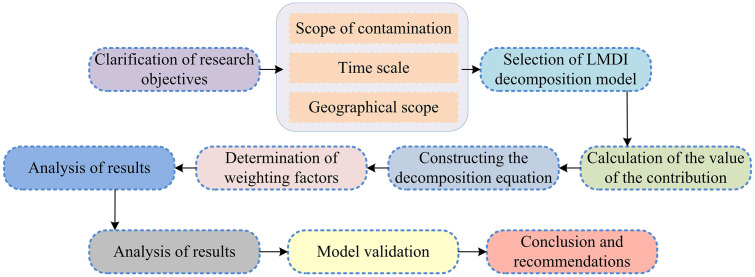
The steps of constructing the model of influencing factors of air pollutant emission based on LMDI decomposition.

In the process of constructing a collaborative ER analysis system for atmospheric pollutants that combines LMDI decomposition and GWR, the primary task is to clarify the driving mechanisms and evolutionary trends of pollutant emissions, and provide theoretical support for regional differentiated collaborative governance strategies. To achieve this goal, it is necessary to construct a decomposition model of emission influencing factors based on LMDI decomposition method, which represents the total amount of pollutant emissions as the product relationship of multiple quantifiable factors [30,31]. The basic process of constructing a model for the influencing factors of atmospheric pollutant emissions based on LMDI decomposition is shown in Fig 2.

**Fig 3:**
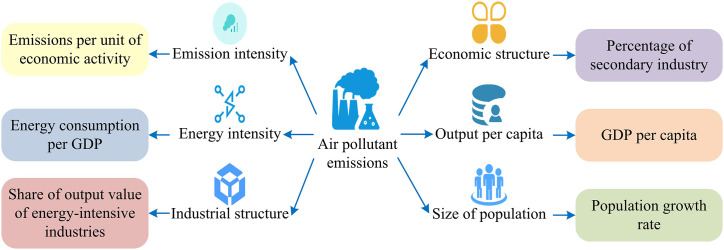
Framework diagram of influencing factors of air pollutant emission.

As shown in Fig 2, the study first selects the appropriate LMDI decomposition model form based on the research problem and the characteristics of the collected data, constructs the decomposition equation [32]. Then, it determines the weight coefficients of each factor, and uses the collected data and the determined weight coefficients to calculate the contribution values of each factor to the changes in atmospheric pollutant emissions. After obtaining the contribution values of each factor, it conducts in-depth analysis of the impact mechanism of each factor on pollutant emissions and explores the interaction relationship. Afterwards, the constructed model is validated to ensure its accuracy and reliability. Finally, based on the model analysis results, targeted policy recommendations are proposed to provide scientific basis for air pollution prevention and control [33,34]. The study first analyzes the temporal driving mechanism of pollutant emissions based on the LMDI method, and decomposes the dynamic changes in emissions into six core driving factors: emission intensity, energy intensity, industrial structure, economic structure, per capita output, and population size, as shown in Fig 3.

**Fig 4:**
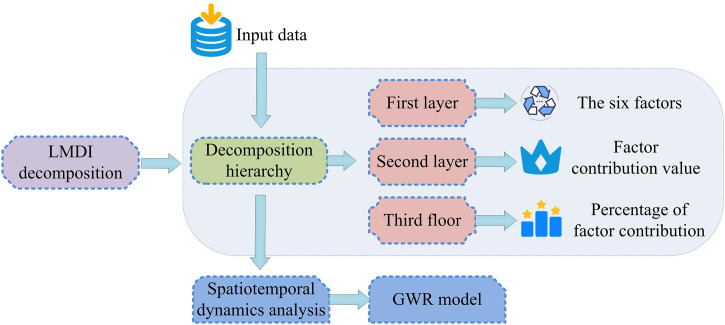
Model of influencing factors of air pollutant emission based on LMDI decomposition.

As shown in Fig 3, emission intensity reflects the level of pollutant emissions caused by unit energy consumption and is a key indicator for measuring technological progress and governance effectiveness. Energy intensity represents the amount of energy consumed per unit of output, reflecting the implementation of energy-saving and ER policies. Industrial structure refers to the proportion of each industry in economic activities, revealing the differences in the contribution of different industries to pollutant emissions. The economic structure further subdivides the economic connections and proportions between industries, emphasizing the driving role of economic transformation in ER. Per capita output is an important indicator for measuring the level of economic development, which directly affects energy demand and total pollution emissions. The population size, as a fundamental factor, determines the overall level of activity and total resource consumption. The study aims to quantitatively reveal the intrinsic mechanism of decoupling economic growth from carbon emissions by comparing the six factors mentioned above, particularly the “per capita output effect” with the “energy intensity effect” and “emission intensity effect”. Among them, the sustained and significant positive “per capita output effect” characterizes a “Extensive Economic Growth” model dominated by factor scale expansion, in which economic growth is highly dependent on the input of production factors such as energy, rather than the improvement of total factor productivity. This model is the core source of pressure that leads to an increase in carbon emissions. On the other hand, the negative “energy intensity effect” and “emission intensity effect” reflect the emission reduction benefits brought about by technological progress and governance improvement. One of the ultimate goals of this model is to quantify the relationship between the growth and decline of these two forces, providing empirical evidence for promoting the transformation of the economy towards an innovation driven, low-carbon intensive, and high-quality development model. The factor analysis model based on LMDI decomposition constructed on this basis is shown in Fig 4.

As shown in Fig 4, the atmospheric pollutant emission influencing factor model based on LMDI decomposition is theoretically based on the extended Kaya identity. A multi-factor decomposition framework consisting of six core driving factors was constructed to adaptively extend the classic model. The model takes the total emissions of atmospheric pollutants as the research object, and then quantitatively calculates the marginal contributions of different factors by introducing the LMDI additive decomposition method, realizing the systematic identification and quantitative decomposition of the driving mechanism of pollutant emission changes, providing data support and analysis basis for subsequent spatial regression modeling and collaborative ER strategy formulation. The expression of Kaya’s identity is shown in equation (1) [35].


$$E=\sum_{i}\left(\frac{E_{i}}{EC_{i}}\times \frac{EC_{i}}{EP_{i}}\times \frac{EP_{i}}{GDP_{i}}\times \frac{GDP_{i}}{GDP}\times \frac{GDP}{P}\times P\right)$$ (1)

In equation (1), i is different regions. E indicates the total amount of pollutant emissions. $E_{i}$ indicates the regional pollution emissions. $EC_{i}$ indicates energy consumption. $EP_{i}$ is energy or economic output. $GDP_{i}$ is the Gross Domestic Product of a region. GDP is the total gross domestic product. P indicates population size. This expression can be reorganized into six main driving factors, as shown in equation (2).


$$E=\sum_{i}\left(EF_{i}\times EI_{i}\times IS_{i}\times ES_{i}\times A\times P\right)$$ (2)

In equation (2), $EF_{i}$ is the unit energy pollutant emission factor. $EI_{i}$ is energy intensity. $IS_{i}$ is industrial structure. $ES_{i}$ is the proportion of industrial economy. A is the per capita level of economic activity. When establishing a pollutant emission decomposition model, it is first necessary to clarify the research time span and spatial distribution boundaries, and select the benchmark year and reporting year for temporal comparative analysis. The total change in pollutant emissions is represented by equation (3).


$$\Delta E=E^{t}-E^{0}$$ (3)

In equation (3), ΔE is the total change in pollutant emissions. $E^{t}$ and $E^{0}$ respectively represent the total emissions of pollutants during the reporting period and the base period. The LMDI method decomposes the changes in pollutant emissions into the contributions of the six driving factors mentioned above, as shown in equation (4).


$$\Delta E=\Delta E_{EF}+\Delta E_{EI}+\Delta E_{IS}+\Delta E_{ES}+\Delta E_{A}+\Delta E_{P}$$ (4)

In equation (4), $\Delta E_{EF}$ is the emission factor effect. $\Delta E_{EI}$ is the energy intensity effect. $\Delta E_{IS}$ is the effect of industrial structure. $\Delta E_{ES}$ is the effects of economic structure. $\Delta E_{A}$ is the per capita output effect. $\Delta E_{P}$ is the population size effect. The contribution calculation formula for each factor is shown in equation (5).


$$\Delta E_{x}=\sum_{i}\left[(E_{i}^{t},E_{i}^{0})\times \ln\left(\frac{x_{i}^{t}}{x_{i}^{0}}\right)\right],x\in \{EF,EI,IS,ES,A,P\}$$ (5)

In equation (5), $E_{i}^{t}$ and $E_{i}^{0}$ represent the emissions of the region i during the reporting period and base period, respectively. $x_{i}^{t}$ and $x_{i}^{0}$ represent the values of the driving factors for the corresponding period.

### 3.3. Establishment of GWR model for collaborative er of atmospheric pollutants

**Fig 5:**
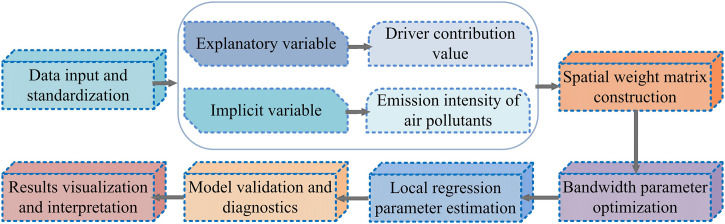
The basic process of building GWR model.

On the basis of clarifying the temporal contributions of six driving factors, including emission intensity, energy intensity, and industrial structure, to atmospheric pollutant emissions, it is essential to further analyze their spatial heterogeneity to support the formulation of collaborative ER strategies. To this end, the contribution values of each factor obtained from LMDI decomposition were used as input variables to construct a GWR model. The differential impact mechanism of different location driving factors on pollution emissions was quantified by introducing spatial weight functions [36]. The bandwidth of the GWR model is determined by minimizing the correction value of the red pool information criterion to ensure the robustness and interpretability of the local regression results. The kernel function selects an adaptive Gaussian kernel to allocate weights reasonably in the case of uneven distribution in the sample space, thereby better revealing spatial differences in different regions. The basic procedure for developing the GWR model is outlined in Fig 5.

As shown in Fig 5, the model takes the pollutant emission intensity of prefecture level cities as the dependent variable and emission intensity, energy intensity, industrial structure, etc. as explanatory variables. It uses an adaptive bandwidth Gaussian kernel function to characterize spatial proximity relationships and generate a local regression coefficient distribution map. Furthermore, it identifies high potential areas for collaboration and conflict areas, and fully presents the spatial pathways of driving factors and the logic of multi-pollutant collaborative optimization. The expression of the local spatial regression model is shown in equation (6) [37].


$$y_{i}=\beta_{0}(u_{i},v_{i})+\sum_{K}^{k=1}\beta_{k}(u_{i},v_{i})x_{ik}+\varepsilon_{i},\;i=1,2,\ldots ,n$$ (6)

**Fig 6:**
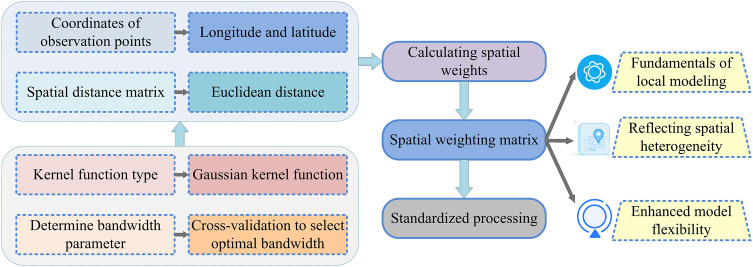
Methods for generating spatial weight matrices.

In equation (6), $y_{i}$ is the atmospheric pollutant emissions or unit emission intensity of the i th spatial unit. $\left(u_{i},v_{i}\right)$ is the spatial coordinates of the i th sample point. $\beta_{k}(u_{i},v_{i})$ indicates the regression coefficient of the variablek at theposition$\left(u_{i},v_{i}\right)$. $\beta_{0}(u_{i},v_{i})$ is the regression constant at the position$\left(u_{i},v_{i}\right)$. $\varepsilon_{i}$ indicates the error term. To estimate the regression coefficients of a certain region, GWR assigns different spatial weights to all sample points. The generation method of spatial weight matrix is shown in Fig 6.

As shown in Fig 6, in the geographically weighted regression model, the study utilizes Gaussian kernel functions to generate spatial weight matrices. Firstly, it is essential to obtain the spatial coordinates of each observation point and calculate their spacing. Subsequently, the appropriate Gaussian kernel function form is selected based on the distance, and the bandwidth parameters are determined through methods such as cross validation. On this basis, the distance and bandwidth values are substituted into a Gaussian function to calculate the spatial weight values point by point, ultimately forming a weight matrix that reflects the degree of spatial proximity influence, providing an accurate and reasonable basis for subsequent local regression analysis. The specific Gaussian kernel function is shown in equation (7) [38].


$$w_{ij}=ecp\left[-{\left(d_{ij}/b\right)}^{2}\right]$$ (7)

In equation (7), $w_{ij}$ is the spatial weight of the i th position relative to the j th position. $d_{ij}$ indicates the distance between the i th and j th spatial points. b is the bandwidth parameter, and it determines the size of the spatial impact range. The setting of spatial weights gives greater weight to observations that are close to the estimated point’s spatial position in model fitting, while samples that are far away from the position have less impact, thus achieving local regression fitting. The bandwidth is determined through cross validation to balance local fitting and model complexity, as shown in equation (8) [39].


$$CV(b)=\sum_{m=1}^{v}{\left(g_{m}-{\hat{g}}_{-m}(b)\right)}^{2}$$ (8)

In equation (8), CV(b) is the cross validation error value calculated under the condition of bandwidth. v indicates the total number of samples. $g_{m}$ indicates the actual value of the m th observation point. ${\hat{g}}_{-m}(b)$ is the predicted value obtained by fitting after removing point i under the condition of bandwidth b. Finally, the bandwidth value that minimizes CV(b) is selected as the optimal bandwidth. Given that local regression in the GWR model may exacerbate the issue of multicollinearity among explanatory variables, particularly between variables such as economic scale, industrial structure, and R&D investment, rigorous diagnosis was conducted at both global and local levels. Firstly, at the global level, calculate the Variance Inflation Factor (VIF) for all explanatory variables. The VIF mean of all variables is below 5, and the tolerance is greater than 0.2, indicating that the overall multicollinearity problem of the model is within an acceptable range. Subsequently, to address the issue of local collinearity, the ridge regression method was employed to optimize the GWR model. By introducing regularization parameters, the estimation of local regression coefficients was effectively stabilized, ensuring the robustness of the model results and the reliability of interpretation.

## 4. Results

### 4.1. Analysis of LMDI decomposition results

To verify the effectiveness and practicality of the constructed framework for collaborative ER of atmospheric pollutants, the study conducted LMDI decomposition analysis and GWR modeling experiments grounded in panel data of Chinese prefecture level cities from 2015 to 2022. The selection of 2022 as the primary data endpoint is due to the fact that official energy, environmental, and economic statistics for 2023 and beyond have not yet been fully released, and there is usually a 1–2 year lag in the approval and disclosure of key indicators. At the same time, the period from 2015 to 2022 fully covers the comprehensive implementation period of the “Action Plan for Air Pollution Prevention and Control” and the implementation effect period of the “Three Year Action Plan for Winning the Blue Sky Defense War”, and extends to the key preliminary stage after the “dual carbon” target is proposed. It can systematically reveal the evolution laws of driving factors in different policy stages and effectively capture the initial trajectory of decoupling economic growth and emissions. The research data was sourced from the China Environmental Statistics Yearbook, MEIC Emission Inventory, China Urban Statistics Yearbook, and the National Basic Geographic Information Center. The study selected Tangshan City as a case study for analysis. The city is an important industrial base located in the Beijing Tianjin Hebei region, with a high proportion of the secondary industry. Its energy structure and industrial transformation path are representative of the region. By extending the Kaya identity and decomposing it using LMDI, the decomposition results of the driving factors of CEs in the city are shown in Table 1.

**Table 1:** Effect decomposition of ce influencing factors in a city from 2015 to 2022.

Year	$\Delta E_{EF}$	$\Delta E_{EI}$	$\Delta E_{IS}$	$\Delta E_{ES}$	$\Delta E_{A}$	$\Delta E_{P}$	ΔE
2015	120.37	−78.92	59.84	40.15	148.63	29.71	319.78
2016	89.64	−109.27	50.32	34.89	139.47	24.83	229.88
2017	−38.51	−152.16	−29.47	19.63	128.94	19.62	−51.95
2018	−59.73	−178.29	−48.91	−9.84	119.82	14.57	−163.38
2019	−80.26	−197.43	−68.35	−24.71	108.59	9.82	−253.34
2020	−99.81	−218.57	−88.74	−39.62	79.34	4.93	−362.47
2021	−69.45	−187.29	−58.16	−29.84	98.73	7.89	−239.12
2022	−48.92	−168.74	−37.59	−14.28	118.46	9.35	−141.72

**Fig 7:**
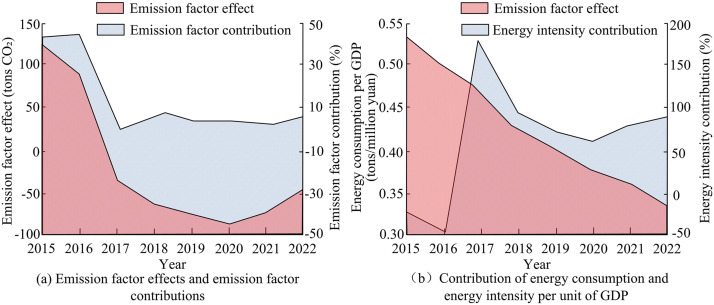
Analysis of contribution rate (CR) of emission intensity and energy intensity factor.

According to Table 1, the total change in carbon emissions for the city shifted from a net increase of 3.1978 million tons in 2015 to a net decrease of 1.4172 million tons in 2022, marking a clear turning point in emissions reduction. The emission intensity effect initially acted as a positive driver, contributing to an increase of 1.2037 million tons in 2015, but turned negative after 2017, resulting in a net reduction of 489,200 tons in 2022. This is directly related to the replacement of coal with natural gas and the promotion of industrial decarbonization technologies. The energy intensity effect has consistently been the core driver of emissions reduction, averaging a reduction of 1.4834 million tons annually, reflecting the sustained inhibitory effect of energy efficiency improvements on CEs. The industrial structure effect turned negative in 2017, with the restructuring of high-energy-consuming industries driving its contribution value from an increase of 598,400 tons to a reduction of 375,900 tons, reflecting the effectiveness of the city’s industrial functional optimization efforts. The per capita output effect has consistently been the largest factor contributing to increased emissions, with an increase of 1.1846 million tons in 2022, indicating that economic development and CEs have not yet been completely decoupled. The population scale effect has weakened year by year, decreasing from an increase of 297,100 tons to an increase of 93,500 tons, but it has consistently had a positive impact on emissions. Affected by the epidemic and Winter Olympics control measures in 2020, the total ER reached 3.6247 million tons, but the subsequent economic recovery led to a rebound in 2022. On this basis, the contribution of factors affecting the CEs of the city was analyzed, starting with emission intensity and energy intensity factors. The outcomes are presented in Fig 7.

**Fig 8:**
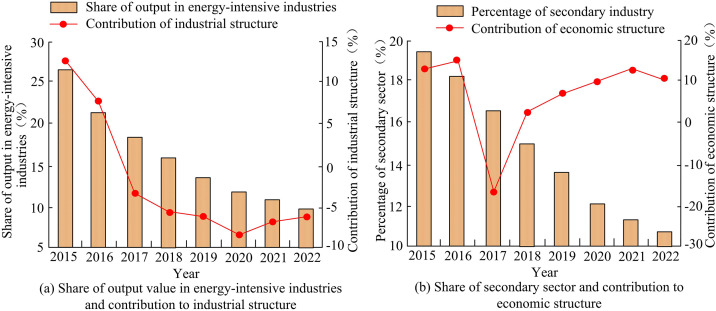
Analysis of CR of industrial structure factor and economic structure factor.

From Fig 7 (a), emission intensity had a promoting effect on the CEs of the city in the early stage, and then changed to a inhibitory effect in the later stage, but the degree of impact was relatively small. In 2017, a key turning point occurred, with the emission factor effect turning negative for the first time −385100 tons, and the CR plummeting to 1.05%. This was attributed to the rapid increase in the proportion of natural gas and the increase in the coverage of ultra-low emission transformation of thermal power, which significantly reduced the CE intensity per unit of energy. By 2022, the promotion of hydrogen powered public transportation and the growth of photovoltaic installed capacity will stabilize the emission factor effect to negative values, but it was still needful to be vigilant about the rebound pressure brought by economic recovery. The curve in Fig 7 (b) shows the changes in the CR of energy consumption and energy intensity per unit of GDP, indicating that the energy intensity effect was the main factor promoting the increase in CEs in the city. The energy consumption per unit of GDP continued to decrease from 0.53 tons/10000 yuan in 2015 to 0.34 tons/10000 yuan in 2022. In 2017, the CR of energy intensity surged abnormally to+185.90%, due to the first decrease in total CEs that year, with energy intensity becoming the dominant factor, corresponding to the popularization of waste heat recovery technology and the upgrading of building energy efficiency standards. After 2017, the CR of energy intensity began to decline, but began to rebound in 2020, indicating that energy efficiency improvement was still the core path for ER. Secondly, the CRs of industrial structure factors and economic structure factors were analyzed, and the outcomes are presented in Fig 8.

**Fig 9:**
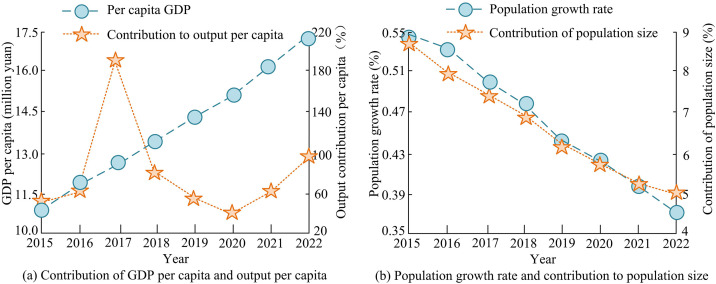
Analysis of CR of per capita output factor and population size factor.

Fig 8 (a) shows the change curve of the proportion of output value of high energy consuming industries and the CR of industrial structure. the proportion of high energy consuming industries in the city’s output value continued to decline from 26.73% in 2015 to 9.75% in 2022, and the CR of industrial structure also exhibited a decreasing tendency. From 2015–2016, industrial structure was a factor that promoted the increase of CEs. Since 2017, the CR of industrial structure turned negative, which had a restraining effect on CEs. The adjustment of industrial structure effectively reduced CE pressure, but it was necessary to continuously optimize the energy efficiency level of emerging industries. According to Fig 8 (b), the proportion of the secondary industry in the city significantly decreased from 19.62% in 2015 to 10.45% in 2022, but the CR of the economic structure showed fluctuations. The CR dropped sharply to −17.79% in 2017, due to the accelerated relocation of high-energy consuming industries and the decrease in CE intensity of the secondary industry. However, after 2018, the CR of economic structure turned positive, reaching +12.85% in 2021, mainly due to the swift advancement of high-end manufacturing industries such as new energy equipment and chip manufacturing. The impact of technological innovation on carbon emissions is negative but weak, possibly due to a significant time lag between R&D investment and emission reduction effects. Specifically, the results of research and development activities usually take several years to translate into actual emission reduction effects, especially when the technology has not yet been widely applied or is still in the experimental stage. In addition, current technological innovations may focus more on improving production efficiency or reducing costs, rather than directly targeting pollution control. This deviation in research and development direction may result in a weaker direct impact on emissions reduction. For example, Y. Xu et al. found in their study of G7 economies that research and development activities have a relatively small impact on CO_2_ emissions in the short term, but have a more significant emission reduction effect in the long term [40]. This indicates that the effectiveness of R&D investment may take a long time to manifest. Meanwhile, the study analyzed the CR of per capita output to population size, as shown in Fig 9.

Fig 9 (a) shows the change curve of the CR between per capita GDP and per capita output. The per capita GDP of the city continued to grow from 108500 yuan in 2015–173600 yuan in 2022, and the per capita output CRshowed significant fluctuations. In 2017, the CR reached an abnormal peak of 192.10%, mainly due to the first decrease in total CEs that year, and the incomplete synergy between per capita output effect and ER policies. In 2020, the CR decreased to 34.76%, mainly due to the slowdown in economic activity caused by the epidemic, with a relatively small per capita GDP growth rate, which partially suppressed the growth of CEs. In 2022, the CR rebounded to 98.68%, corresponding to a 17.16% increase in per capita GDP after economic recovery, indicating that economic growth remained the core driver of CEs. Based on Fig 9 (b), the population growth rate gradually decreased from 0.54% in 2015 to 0.37% in 2022, and the CR of population size synchronously decreased from 8.89% to 4.98%, reflecting the promoting effect of population size on CEs. Although the population size effect was weakening year by year, resource agglomeration still exerted rigid pressure on CEs, and its impact needed to be further reduced through spatial optimization and low-carbon employment structure transformation. To further explore the long-term relationship between economic development and CE pressure, the study evaluated the decoupling status of economic growth and carbon emissions in Tangshan City from 2015 to 2022 based on the Tapio decoupling model. The specific results are shown in Table 2.

**Table 2:** Tapio decoupling analysis of economic growth and CEs in a city (2015–2022).

Year	CE change rate (%)	GDP change rate (%)	Elastic index (e)	Decoupling state
2015–2016	8.4	12.1	0.69	Weak decoupling
2016–2017	−12.5	6.8	−1.84	strong decoupling
2017–2018	−18.2	5.1	−3.57	strong decoupling
2018–2019	−15.2	4.3	−3.53	strong decoupling
2019–2020	−19.8	−2.1	9.43	Recessionary decoupling
2020–2021	9.5	8.2	1.16	Expansionary negative decoupling
2021–2022	−8.2	5.5	−1.49	strong decoupling
2015–2022	−28.5	42.7	−0.67	strong decoupling

According to Table 2, during the research period, the city as a whole achieved a strong decoupling between economic development and carbon emissions. While the cumulative economic growth was 42.7%, the total carbon emissions significantly decreased by 28.5%, with an elasticity index of −0.67. However, the effectiveness of this decoupling needs to be carefully evaluated in conjunction with the evolution of economic growth rates. As shown in Table 2, the annual GDP growth rate of the city has shown a fluctuating downward trend from 12.1% in 2015–2016, and has dropped to 4.3% in 2018–2019. In 2020, there was a negative growth of 2.1% due to the epidemic. The gradual slowdown of economic growth momentum objectively creates a favorable macro environment for emission reduction and reduces the pressure of new emissions. The continuous strong decoupling from 2017 to 2019 was undoubtedly due to the mandatory adjustment of industrial and energy structure driven by strict environmental policies, but the sustained slowdown in economic growth from 6.8% to 4.3% during the same period is also a factor that cannot be ignored. The recessionary decoupling in 2020 was entirely dominated by abnormal contraction of economic activity. What is particularly alarming is that in 2021, the economic growth rate rebounded strongly to 8.2%, immediately triggering an expansionary negative decoupling. This strongly indicates that the city’s economic growth and carbon emissions have not yet achieved an inherent fundamental decoupling, and its decoupling status is still significantly affected by economic cycle fluctuations, rather than being driven entirely by green and low-carbon transformation. Until 2022, with a moderate economic growth rate of 5.5%, the strong decoupling will be re achieved, which more reliably confirms the effectiveness of the “green recovery” policy. According to Table 2, the current decoupling achievements still have certain fragility and external dependence. Consolidating the long-term decoupling mechanism cannot rely on economic slowdown. The key is to go beyond short-term administrative intervention, deeply integrate green and low-carbon into long-term economic growth drivers and industrial structure optimization, and promote a stable transformation from “relative decoupling driven by slowing growth rate” to “absolute decoupling driven by high-quality development”.

### 4.2. GWR result analysis

Based on the decomposition results of LMDI, a GWR model was constructed with a bandwidth of 120 km. Firstly, taking the Beijing Tianjin Hebei (BTH) region as an example, the synergistic ER effects of five major atmospheric pollutants, CO, CO_2_, SO_2_, NOx, and PM_2.5_, were analyzed. The outcomes are presented in Table 3.

**Table 3:** Analysis of the effect of coordinated ERof air pollutants (2015–2022).

Pollutant mix	SO_2_-PM_2.5_	NOx-PM_2.5_	CO_2_-SO_2_	CO_2_-NOx	PM_2.5_-CO
Collaborative ER ratio	1:0.38	1:0.21	1:0.12	1:0.09	1:0.05
Average annual synergistic ER (tonnes)	29.7	18.2	12.5	8.7	3.2
Regional coordination index	0.85	0.72	0.58	0.49	0.33
Significance (*p* value)	<0.01	0.03	0.12	0.21	0.45
Main driver correlation	Emission factor(R = 0.76)	Energy intensity(R = 0.63)	Industrial structure (R = 0.41)	Economic structure(R = 0.35)	Population size(R = 0.18)

**Fig 10:**
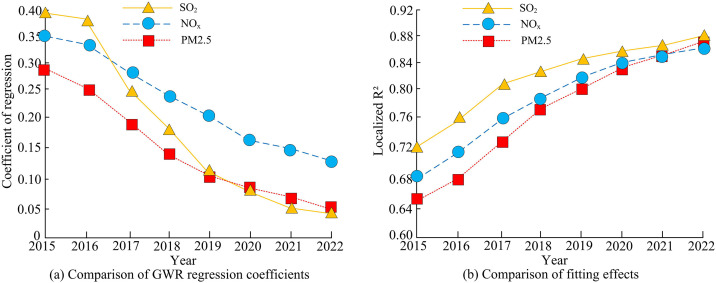
Comparison between GWR regression coefficient and local R2 in Beijing-Tianjin-Hebei region.

From Table 3, the synergistic ER ratio of SO2-PM_2.5_was 1: 0.38, with an average annual ER of 297000 tons, a regional synergy index of 0.85, and a *p*-value less than 0.01, indicating strong significance. In contrast, the ratio of NOx PM_2.5_ was only 1:0.21, with an average annual ER of 182000 tons, a regional synergy index of 0.72, and *a p*-value of 0.03, indicating a weaker effect. This difference mainly stemmed from the differences in pollutant generation mechanisms and policy implementation efficiency. SO_2_ is an important precursor of PM_2.5_, and the sulfate particles generated by its oxidation directly contribute to PM_2.5_ concentration. Therefore, desulfurization measures such as ultra-low emission transformation of coal-fired power plants can efficiently achieve dual ER. The treatment of NOx involved mobile sources such as diesel vehicles and ships, as well as dispersed industrial sources, which were technically complex and difficult to coordinate. In addition, SO_2_ER relied on optimizing emission factors (R = 0.76) and reducing energy intensity (R = 0.63), with direct and significant measures. The reduction of NOx emissions relied more on industrial structure upgrading (R = 0.41) and economic structure adjustment (R = 0.35), and progress lags behind. The synergistic emission reduction effects of NOx-related pollutants are relatively weak, primarily due to the dispersed distribution of their emission sources and the complexity of treatment technologies. NOx emissions originate from industrial combustion, transportation, shipping, and dispersed industrial sources, making control challenging. Policy implementation and technology promotion require more time and regional coordination. Additionally, secondary particulate matter generated from NOx is significantly influenced by meteorological conditions, further increasing the uncertainty of emission reductions. Therefore, even if measures such as industrial structure optimization or economic structure adjustment are implemented, their short-term emission reduction effects remain limited. Comprehensive strategies are needed, including transportation electrification, clean energy substitution for mobile sources, and cross-regional collaborative governance. Meanwhile, the study evaluated the implementation effect of air pollution control policies in the BTH region, analyzed the correlation characteristics and spatial heterogeneity between major pollutants and CO_2_ emissions, and the outcomes are presented in Fig 10.

**Fig 11:**
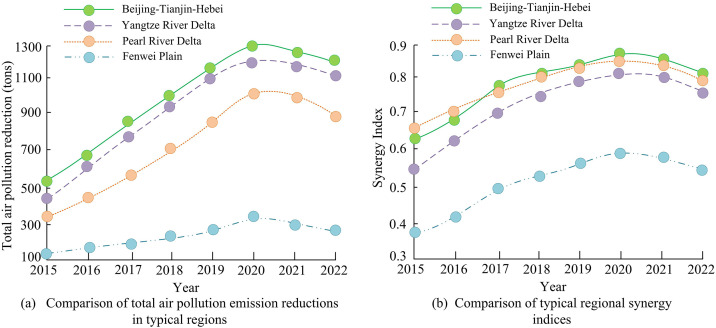
Comparison of total ER and synergistic index of typical regional air pollutants.

Fig 10 (a) shows the regression coefficient changes of atmospheric pollutants and CO_2_ emissions in the BTH region from 2015 to 2022. The SO_2_ coefficient decreased from 0.39 to 0.03, a decrease of 92.31%, mainly due to the substitution of coal and the ultra-low emission transformation of thermal power. After 2017, policy efforts increased and the proportion of natural gas increased significantly. The NOx coefficient decreased from 0.35 to 0.13, a decrease of 62.86%, reflecting the phasing out of diesel vehicles and the application of denitrification technology, but the growth in transportation demand dragged down the speed of ER. The PM_2.5_ coefficient decreased from 0.28 to 0.05, a decrease of 82.14%, benefiting from the upgrade of dust removal technology. However, the decrease in secondary particulate matter slowed down later due to meteorological factors. Fig 10 (b) shows the improved local explanatory power of the GWR model for pollutants. The R² of SO_2_ increased from 0.72 to 0.88, due to significant differences in the effectiveness of high-energy consumption industry relocation policies in Tangshan, Shijiazhuang and other places. The R² of NOx increased from 0.68 to 0.86, and traffic source control and port cleaning gradually covered, but the dispersion of mobile sources caused fluctuations in some areas. The R^2^ of PM2.5 increased from 0.65 to 0.87, indicating an enhanced analysis of the secondary generation mechanism by the model, especially the strengthened correlation path between industrial production restrictions and joint prevention and control policies during the 2020 Winter Olympics. The overall R² growth confirmed that GWR could accurately capture spatial heterogeneity, but relied on high-quality geographic data support. On this basis, a comparison was made between the total ER and synergy index of the four typical regions of BTH, Yangtze River Delta (YRD), Pearl River Delta (PRD), and Fenwei Plain (FP) to verify the stability of the GWR model. The outcomes are presented in Fig 11.

According to Fig 11 (a), the ER of 13.5 million tons in the BTH region ranked first in 2020, and decreased to 12.1 million tons in 2022. The resumption of steel production and the rebound in transportation had a drag effect. The PRD reduced emissions by 8.9 million tons in 2022, with the service industry seeing its peak in emissions reduction and industrial decarbonization slowing down. The FP only produced 2.6 million tons in 2022, with a rebound in steel production capacity and inefficient dust removal technology. According to Fig 11 (b), the synergy index of BTH reached its peak of 0.88 in 2020, driven by strong measures such as inter provincial production restrictions and data sharing during the Winter Olympics. In 2022, it fell back to 0.83, reflecting the rebound of some high energy consuming industries after economic recovery, and the weakening of synergy. The synergy index between the YRD and the PRD steadily increased to 0.77 and 0.81, benefiting from the promotion of carbon trading and the unification of technical standards. The FPwas always below 0.6, indicating that the region needed to strengthen inter provincial governance to improve ER effectiveness. Finally, to verify the accuracy of the GWR model, a comparison was made between the OLS model and the Spatial Autoregressive Regression (SAR) model. The outcomes are presented in Table 4.

**Table 4:** Performance comparison of GWR model with other spatial regression models.

Model fitting and diagnostic statistics	Coefficient of determination (R^2^)	Adjusted R^2^	Correction criteria for Akaike information content (AICc)	Residual Sum of Squares (RSS)	Residual Morans I index	Proportion of significant variables(*p* < 0.05)
GWR model	0.86	0.83	132.54	45.73	0.07	0.75
OLS model	0.72	0.69	158.70	76.28	0.25	0.37
SAR model	0,78	0.75	145.22	56.32	0.15	0.51

According to Table 4, the R^2^ of the GWR model was 0.86, and after adjustment, the R^2^ reached 0.83, significantly higher than the 0.69 of the OLS model and the 0.75 of the SAR model. This indicated that it could more accurately capture data variability, especially when considering spatial heterogeneity, with significant advantages. The Red Pool Information Content (AICc) corrected by the GWR model decreased by an average of 12.78% compared to other models. The sum of squared residuals (RSS) of GWR was 45.73, much lower than the 76.28 of OLS and 56.32 of SAR, indicating that its prediction error was smaller and its accuracy was higher. The residual Morans I index was 0.07, close to zero and not significant, indicating that GWR effectively eliminated the spatial autocorrelation of residuals. However, the residuals of OLS and SAR still exhibited significant spatial dependence, with indices of 0.25 and 0.15, respectively, leading to a decrease in model reliability. In terms of the proportion of significant variables, GWR reached 75%, far exceeding OLS’s 37.5% and SAR’s 51%, as it identified more region specific driving factors through local regression, such as the sensitivity of energy intensity in the BTH region and the potential for technological upgrading in the YRD.

## 5. Discussion and conclusion

In response to the challenge of insufficient spatial heterogeneity analysis in regional air pollution collaborative governance, a research framework combining LMDI decomposition and GWR was constructed to identify multi factor driving mechanisms and regional ER potential. Decompose emission changes into six major factors through LMDI decomposition, revealing the temporal driving mechanism;Then, using the GWR model, a spatial weight matrix was constructed to quantify the spatial differences in factor effects. Ultimately, combining the interaction of multiple pollutants, the collaborative ER path was optimized. The total change in carbon emissions in Tangshan City has gradually shifted from a net increase of 3.1978 million tons in 2015 to a net decrease of 1.4172 million tons in 2022, with energy intensity and emission intensity effects being the dominant factors in the city’s ER. The collaborative ER analysis in the BTH region showed that the annual collaborative ER of SO2-PM2.5 combination reached 297000 tons and had strong significance, while the effect of NOx related combination was relatively weak. In addition, the R2 (0.86) and residual control (Morans I = 0.07) of the GWR model were both superior to traditional methods. The results indicated that multi-pollutant synergy required priority optimization of emission intensity and energy structure, while suppressing extensive economic growth. Regions such as BTH could strengthen collaboration through industrial relocation and energy substitution, while the FP needed to break through technological bottlenecks and cross regional governance. In addition, The role of industrial structure in pollutant emissions exhibits significant regional heterogeneity. In coastal areas with a high proportion of service industry, the increase in added value of the tertiary industry can effectively reduce unit energy consumption and emission levels, thereby suppressing SO2, NOx, and PM2.5 emissions. In heavy industry or resource-based central and western provinces, the upgrading of industrial structure often accompanies the expansion of high energy consuming industries such as coal chemical and metallurgical industries in the short term, thereby promoting pollutant emissions [41,42]. This complex effect indicates that the emission reduction effect of industrial restructuring depends on the regional economic foundation and industrial development path, and policy formulation needs to be combined with regional characteristics to promote the development of the service industry and the green transformation of heavy industry. The limitation of the research was that it did not consider the dynamic interference of meteorological factors on spatial weights. In the future, machine learning can be introduced to optimize factor weight allocation and explore the ER effects of multi policy coupling under the “dual carbon” target, in order to enhance the model’s foresight and further optimize the formulation and implementation of collaborative ER strategies.

## Supporting information

S1 File Minimal Data Set Definition. (DOC)
