# Supplementary material for: Decomposing drivers of air pollutant emissions in China: A hybrid LMDI and Geographically Weighted Regression approach
Source: PLoS One. 2025 Oct 21;20(10):e0333898. doi: 10.1371/journal.pone.0333898 (PMC12539709; doi:10.1371/journal.pone.0333898)
Supplement: S1 File — (DOC) [file pone.0333898.s001.doc]

**Table 1. Effect decomposition of ce influencing factors in a city from 2015 to 2022**

| Year | $\text{Δ}E_{EF}$ | $\text{Δ}E_{EI}$ | $\text{Δ}E_{IS}$ | $\text{Δ}E_{ES}$ | $\text{Δ}E_{A}$ | $\text{Δ}E_{P}$ | $\text{Δ}E$ |
| --- | --- | --- | --- | --- | --- | --- | --- |
| 2015 | 120.37 | -78.92 | 59.84 | 40.15 | 148.63 | 29.71 | 319.78 |
| 2016 | 89.64 | -109.27 | 50.32 | 34.89 | 139.47 | 24.83 | 229.88 |
| 2017 | -38.51 | -152.16 | -29.47 | 19.63 | 128.94 | 19.62 | -51.95 |
| 2018 | -59.73 | -178.29 | -48.91 | -9.84 | 119.82 | 14.57 | -163.38 |
| 2019 | -80.26 | -197.43 | -68.35 | -24.71 | 108.59 | 9.82 | -253.34 |
| 2020 | -99.81 | -218.57 | -88.74 | -39.62 | 79.34 | 4.93 | -362.47 |
| 2021 | -69.45 | -187.29 | -58.16 | -29.84 | 98.73 | 7.89 | -239.12 |
| 2022 | -48.92 | -168.74 | -37.59 | -14.28 | 118.46 | 9.35 | -141.72 |
| 2023 | -65.18 | -190.25 | -52.41 | -20.15 | 105.83 | 8.71 | -213.45 |
| 2024 | -82.07 | -212.88 | -66.34 | -25.93 | 95.17 | 7.89 | -284.16 |

**Table.2 Tapio decoupling analysis of economic growth and CEs in a city (2015-2022)**

| Year | CE change rate (%) | GDP change rate (%) | Elastic index (e) | Decoupling state |
| --- | --- | --- | --- | --- |
| 2015-2016 | 8.4 | 12.1 | 0.69 | Weak decoupling |
| 2016-2017 | -12.5 | 6.8 | -1.84 | strong decoupling |
| 2017-2018 | -18.2 | 5.1 | -3.57 | strong decoupling |
| 2018-2019 | -15.2 | 4.3 | -3.53 | strong decoupling |
| 2019-2020 | -19.8 | -2.1 | 9.43 | Recessionary decoupling |
| 2020-2021 | 9.5 | 8.2 | 1.16 | Expansionary negative decoupling |
| 2021-2022 | -8.2 | 5.5 | -1.49 | strong decoupling |
| 2015-2022 | -28.5 | 42.7 | -0.67 | strong decoupling |

**Table 3. Analysis of the effect of coordinated ERof air pollutants (2015-2022)**

| Pollutant mix | SO_2_-PM_2.5_ | NOx-PM_2.5_ | CO_2_-SO_2_ | CO_2_-NOx | PM_2.5_-CO |
| --- | --- | --- | --- | --- | --- |
| Collaborative ER ratio | 1:0.38 | 1:0.21 | 1:0.12 | 1:0.09 | 1:0.05 |
| Average annual synergistic ER (tonnes) | 29.7 | 18.2 | 12.5 | 8.7 | 3.2 |
| Regional coordination index | 0.85 | 0.72 | 0.58 | 0.49 | 0.33 |
| Significance (*p* value) | <0.01 | 0.03 | 0.12 | 0.21 | 0.45 |
| Main driver correlation | Emission factor(R=0.76) | Energy intensity(R=0.63) | Industrial structure (R=0.41) | Economic structure(R=0.35) | Population size(R=0.18) |

**Table 4. Performance comparison of GWR model with other spatial regression models**

| Model fitting and diagnostic statistics | Coefficient of determination (R^2^) | Adjusted R^2^ | Correction criteria for Akaike information content (AICc) | Residual Sum of Squares (RSS) | Residual Morans I index | Proportion of significant variables(*p*<0.05) |
| --- | --- | --- | --- | --- | --- | --- |
| GWR model | 0.86 | 0.83 | 132.54 | 45.73 | 0.07 | 0.75 |
| OLS model | 0.72 | 0.69 | 158.70 | 76.28 | 0.25 | 0.37 |
| SAR model | 0,78 | 0.75 | 145.22 | 56.32 | 0.15 | 0.51 |

**Table 5. Fig.7 raw data**

| Indicator | Emission factor effect (tons CO₂) | Emission factor contribution (%) | Energy consumption per GDP  (tons/million yuan) | Energy intensity contribution (%) |
| --- | --- | --- | --- | --- |
| 2015 | 123.78 | 43.06 | 0.53 | -17.01 |
| 2016 | 88.81 | 44.44 | 0.50 | -46.79 |
| 2017 | -38.51 | 1.05 | 0.47 | 185.90 |
| 2018 | -65.03 | 5.56 | 0.42 | 91.59 |
| 2019 | -77.27 | 3.47 | 0.40 | 70.51 |
| 2020 | -87.76 | 4.17 | 0.37 | 58.13 |
| 2021 | -75.52 | 2.08 | 0.36 | 78.70 |
| 2022 | -49.30 | 6.25 | 0.34 | 87.16 |

**Table 6. Fig.8 raw data**

| Indicator | Share of output in energy-intensive industries（%） | Contribution of industrial structure（%） | Percentage of secondary sector（%） | Contribution of economic structure（%） |
| --- | --- | --- | --- | --- |
| 2015 | 26.73 | 12.71 | 19.62 | 13.31 |
| 2016 | 21.37 | 7.7 | 18.24 | 15.07 |
| 2017 | 18.20 | -3.31 | 16.55 | -17.79 |
| 2018 | 15.91 | -5.77 | 14.93 | 2.39 |
| 2019 | 13.45 | -6.30 | 13.59 | 7.32 |
| 2020 | 11.69 | -8.59 | 12.04 | 10.14 |
| 2021 | 10.81 | -6.83 | 11.20 | 12.85 |
| 2022 | 9.75 | -6.30 | 10.45 | 10.49 |

**Table 7. Fig.9 raw data**

| Indicator | GDP per capita (million yuan) | Output contribution per capita（%） | Population growth rate (%) | Contribution of population size (%) |
| --- | --- | --- | --- | --- |
| 2015 | 10.85 | 52.45 | 0.54 | 8.89 |
| 2016 | 11.82 | 64.30 | 0.53 | 7.96 |
| 2017 | 12.65 | 192.10 | 0.50 | 7.42 |
| 2018 | 13.47 | 80.23 | 0.47 | 6.89 |
| 2019 | 14.34 | 54.67 | 0.44 | 6.15 |
| 2020 | 15.15 | 34.76 | 0.43 | 5.68 |
| 2021 | 16.21 | 61.33 | 0.39 | 5.21 |
| 2022 | 17.36 | 98.68 | 0.37 | 4.98 |

**Table 8. Fig.10 raw data**

| Indicator | Coefficient of regression | | | Localized R² | | |
| --- | --- | --- | --- | --- | --- | --- |
| Pollutant type | SO_2_ | NO_x_ | PM2.5 | SO_2_ | NO_x_ | PM2.5 |
| 2015 | 0.39 | 0.35 | 0.28 | 0.72 | 0.68 | 0.65 |
| 2016 | 0.38 | 0.33 | 0.25 | 0.76 | 0.72 | 0.68 |
| 2017 | 0.25 | 0.28 | 0.19 | 0.81 | 0.76 | 0.73 |
| 2018 | 0.18 | 0.24 | 0.14 | 0.83 | 0.79 | 0.78 |
| 2019 | 0.11 | 0.21 | 0.10 | 0.85 | 0.82 | 0.81 |
| 2020 | 0.08 | 0.17 | 0.08 | 0.86 | 0.84 | 0.84 |
| 2021 | 0.05 | 0.15 | 0.06 | 0.87 | 0.85 | 0.86 |
| 2022 | 0.03 | 0.13 | 0.05 | 0.88 | 0.86 | 0.87 |

**Table 9. Fig.11 raw data**

| Indicator | Region Name | 2015 | 2016 | 2017 | 2018 | 2019 | 2020 | 2021 | 2022 |
| --- | --- | --- | --- | --- | --- | --- | --- | --- | --- |
| Total air pollution reduction (tons) | Beijing-Tianjin-Hebei | 535.23 | 678.24 | 870.98 | 1013.99 | 1175.65 | 1350.00 | 1256.48 | 1210.17 |
|  | Yangtze River Delta | 441.97 | 616.06 | 790.15 | 951.82 | 1113.47 | 1206.74 | 1181.87 | 1132.12 |
|  | Pearl River Delta | 336.27 | 441.97 | 566.32 | 721.76 | 870.98 | 1038.96 | 1013.99 | 890.29 |
|  | Fenwei Plain | 110.43 | 147.84 | 189.46 | 224.73 | 286.54 | 347.90 | 300.05 | 260.16 |
| Synergy Index | Beijing-Tianjin-Hebei | 0.63 | 0.69 | 0.79 | 0.82 | 0.84 | 0.88 | 0.86 | 0.83 |
|  | Yangtze River Delta | 0.54 | 0.62 | 0.71 | 0.75 | 0.79 | 0.82 | 0.80. | 0.77 |
|  | Pearl River Delta | 0.66 | 0.71 | 0.76 | 0.81 | 0.84 | 0.86 | 0.84 | 0.81 |
|  | Fenwei Plain | 0.37 | 0.42 | 0.50 | 0.53 | 0.57 | 0.59 | 0.58 | 0.55 |
